# Innovative tree-based method for sampling molecular conformations: exploring the ATP-binding cassette subfamily D member 1 (ABCD1) transporter as a case study

**DOI:** 10.3389/fmolb.2024.1440529

**Published:** 2024-08-01

**Authors:** Thomas Haschka, Foudil Lamari, Fanny Mochel, Violetta Zujovic

**Affiliations:** ^1^ Sorbonne Université, Institut du Cerveau - Paris Brain Institute - ICM, Inserm, CNRS, APHP, Hôpital Pitié la Salpétrière University Hospital, DMU Neuroscience 6, Paris, France; ^2^ UF Biochimie des Maladies Neuro-métaboliques, Service de Biochimie Métabolique, APHP, Hôpital Pitié la Salpétrière University Hospital, Paris, France

**Keywords:** molecular simulation, tree, ABCD1, transporter, membrane, adrenoleukodystrophy, XAld, conformational space sampling

## Abstract

We introduce a novel tree-based method for visualizing molecular conformation sampling. Our method offers enhanced precision in highlighting conformational differences and facilitates the observation of local minimas within proteins fold space. The projection of empirical laboratory data on the tree allows us to create a link between protein conformations and disease relevant data. To demonstrate the efficacy of our approach, we applied it to the ATP-binding cassette subfamily D member 1 (ABCD1) transporter responsible for very long-chain fatty acids (VLCFAs) import into peroxisomes. The genetic disorder called X-linked adrenoleukodystrophy (XALD) is characterized by the accumulation of VLCFA due to pathogenic variants in the ABCD1 gene. Using *in silico* molecular simulation, we examined the behavior of 16 prevalent mutations alongside the wild-type protein, exploring both inward and outward open forms of the transporter through molecular simulations. We evaluated from resulting trajectories the energy potential related to the ABCD1 interactions with ATP molecules. We categorized XALD patients based on the severity and progression of their disease, providing a unique clinical perspective. By integrating this data into our numerical framework, our study aimed to uncover the molecular underpinnings of XALD, offering new insights into disease progression. As we explored molecular trajectories and conformations resulting from our study, the tree-based method not only contributes valuable insights into XALD but also lays a solid foundation for forthcoming drug design studies. We advocate for the broader adoption of our innovative approach, proposing it as a valuable tool for researchers engaged in molecular simulation studies.

## 1 Introduction

We present here a novel tree-based method for molecular conformation sampling. Our study revolves around the ATP-binding cassette subfamily D member 1 (ABCD1) transporter, central to X-linked adrenoleukodystrophy (XALD) pathogenesis.

The function of the ABCD1 transporter is to carry very long chain fatty acid (VLCFA) consisting of 22 or more carbon atoms, across the membrane from the cytosol into the peroxisome. Failure due to mutation or absence of the protein causes an accumulation of VLCFA in plasma as well as in tissues. This includes the adrenal cortex, the spinal cord and the white matter of the brain (Moser et al., 1981). The disease caused by such a dysfunction effects all parts of the globe (Kemp et al., 2001), and has an estimate prevalence of 1 in 17,000 newborns (Bezman and Moser, 1998). Besides VLCFA transport deficiency it was shown that an interplay with mitochondrial dynamics plays a role in the progression of the disease (Launay et al., 2024).

In this study we delve into 16 mutations of ABCD1 prevalent in a reference center patient population. The phenotypic variability of the patients is ranging from a devastating inflammatory childhood cerebral adrenoleukodystrophy (C-CALD) affecting boys to a progressive spastic paraplegia in adulthood (adrenomyeloneuropathy, AMN) affecting both adult men and women. Over the past decade, it appears that the majority of AMN men – 50% over 10 years (1) – will also develop CALD later in life (A-CALD) with the same grim prognosis as children (Raymond et al., 1999; Berger et al., 2014; Turk et al., 2020). Over 800 mutations have been identified in the ABCD1 coding region, yet no clear link between specific mutations and phenotypic outcomes has been established (Palakuzhiyil et al., 2020). Notably, even twins with identical mutations can exhibit different forms of the disease (Palakuzhiyil et al., 2020). Despite this challenge, with the molecular structure of ABCD1 now accessible (Chen et al., 2022), we are actively investigating the mutations found in our patients in-house in an effort to understand how these mutations correlate with observed disease progressions.

The primary objective is to establish a structure-function relationship among these disease types which are characterized by differences in disease progression. Our approach involves *in silico* modeling of the ABCD1 protein in both inward and outward open states, generating models for wild type and mutations inserted into a modeled membrane. Molecular dynamics simulations produce trajectories for wild type and mutations, aiming to correlate molecular conformational changes with specific disease progressions.

Research in visualizing molecular trajectories and extracting meaningful insights from the vast data they encompass is actively evolving. Various approaches exist for visualizing these trajectories directly through different tools, as reviewed in (Belghit et al., 2024). Popular molecular dynamics suites like GROMACS (Abraham et al., 2015) or NAMD (Phillips et al., 2005), often used in conjunction with VMD (Humphrey et al., 1996), offer diverse tools for analyzing molecular trajectories. Python libraries such as MDAnalysis (Michaud-Agrawal et al., 2011; Richard J.; Gowers et al., 2016) have been developed specifically to analyze and graphically represent data from these trajectories. Despite these advances, the challenge of efficiently summarizing the often extensive number of molecular conformations persists. Trajectory mapping, for example, attempts to visualize entire molecular simulation datasets in a single graph (Kožić and Bertoša, 2024). Another significant advancement is the application of the DBSCAN algorithm (Ester et al., 1996) to locate local energy minima within molecular trajectories and conformational spaces (Liu et al., 2021). Inspired by our prior work on MNHN-Tree-Tools (Haschka et al., 2021), where we applied DBSCAN to cluster gene sequences adaptively and hierarchically, we have adapted MNHN-Tree-Tools for similar tasks in molecular dynamics. This adaptation allows us to leverage tree visualizations to explore and interpret molecular dynamics trajectories, as detailed in this study.

The algorithm identifies clusters of conformations that are densely connected within a space sampled by molecular dynamics simulations. This is determined by the condition:

$$\rho_{L}>\frac{\text{minpts}}{V\left(\epsilon_{L}\right)},$$ (1)

where ρL represents the density of clusters at iteration L, and the right-hand side reflects the minimum density requirement set by the DBSCAN algorithm. Here, minpts denotes the number of conformations expected within a hyperdimensional sphere of volume V and radius ε. These clusters typically occupy a subspace of the overall conformational space defined by principal components from PCA. In contrast to traditional DBSCAN, this algorithm introduces a third parameter Δε, which incrementally expands ε and triggers additional DBSCAN runs after each increment. As ε increases, the volume in Eq. 1 expands leading to lower densities and the identification of more diffuse clusters in each iteration. For a subsequent layer (iteration) of the tree L+1 it follows Eq. 2:

$$\rho_{L+1}>\frac{\text{minpts}}{V\left(\epsilon_{L}+\Delta \epsilon \right)}.$$ (2)

Alternatively, we can express ε_L_ = ε_L−1_ + Δε.

**FIGURE 1:**
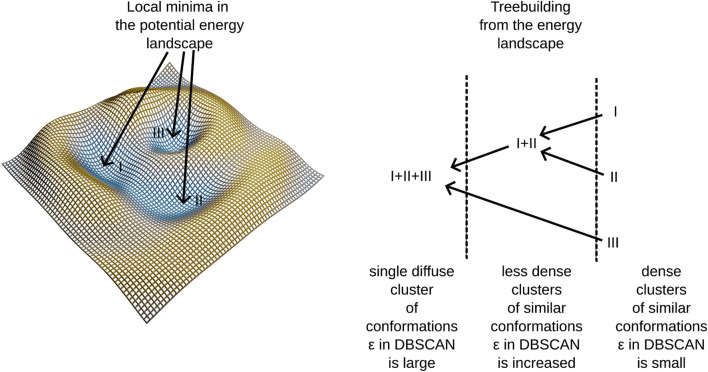
An illustration of treebuilding from a potential energy landscape.

In our previous work Haschka et al. (2021), we demonstrated that using this algorithm allows us to construct a hierarchical tree structure by embedding dense clusters into progressively more diffuse clusters until all elements of the conformational space merge into a single cluster—the root of the tree. We also noted that dense clusters tend to form in regions corresponding to potential energy wells within the force field employed in molecular dynamics simulations. For a clearer understanding of the tree-building process, refer to the schema presented in Figure 1 and the algorithmic supplement of (Haschka et al., 2021).

This method identifies clusters in the conformational space, building a tree linking different potential energy wells. Notably, certain branches into different energy-wells are found to be disease-progession-specific, linking molecular conformations to patient observations. A further description of our algorithm is provided in Section 2.6. The algorithm employs adaptive density clustering, aided by principal component analysis in order to analyze high-dimensional conformational states. The resulting *density to energy well* correspondence reveals insights into the energy landscape, providing a more nuanced understanding of molecular dynamics trajectories.

Beyond XALD, we propose our tree-based method as a versatile tool for researchers in molecular simulation studies, applicable to diverse molecular systems. Our study not only uncovers structural implications of ABCD1 mutations but also could serve for future drug design studies around XALD and related membrane protein mutations.

In brief, our investigation unveils the intricate relationship between specific ABCD1 mutations and XALD form of disease progression, leveraging a novel tree-based conformational sampling approach. Our results contribute to understanding structure-function relationships, paving the way for future drug design studies, and advocate for the broader adoption of our method in molecular dynamics research.

## 2 Methods

### 2.1 Molecular modelling

To facilitate molecular dynamics simulations, two distinct molecular models of the wild-type ABCD1 transporter were created, representing the cytosol-open and peroxisome-open structures. The Protein Data Bank (PDB) structure with accession code 7VWC (Chen et al., 2022) was used as a template to model the cytosol-open structure. Missing residues were inserted using the MODELLER (Eswar et al., 2006) software. However, for the peroxisome-open structure, a more elaborate process was required. The PDB structure 7VX8 (Chen et al., 2022) was used as a template, but MODELLER alone produced unpromising models. To complete the missing residues (346–382 and 436–460), parts of the protein structure from 7VWC and the AlphaFold (Jumper et al., 2021; Varadi et al., 2021) prediction for human ABCD1 were aligned, and MODELLER was used in order to build the final wild type template protein structure from this alignment. The resulting peroxisome-open structure included ATP molecules as found in 7VX8 and were used in molecular simulations.

**FIGURE 2:**
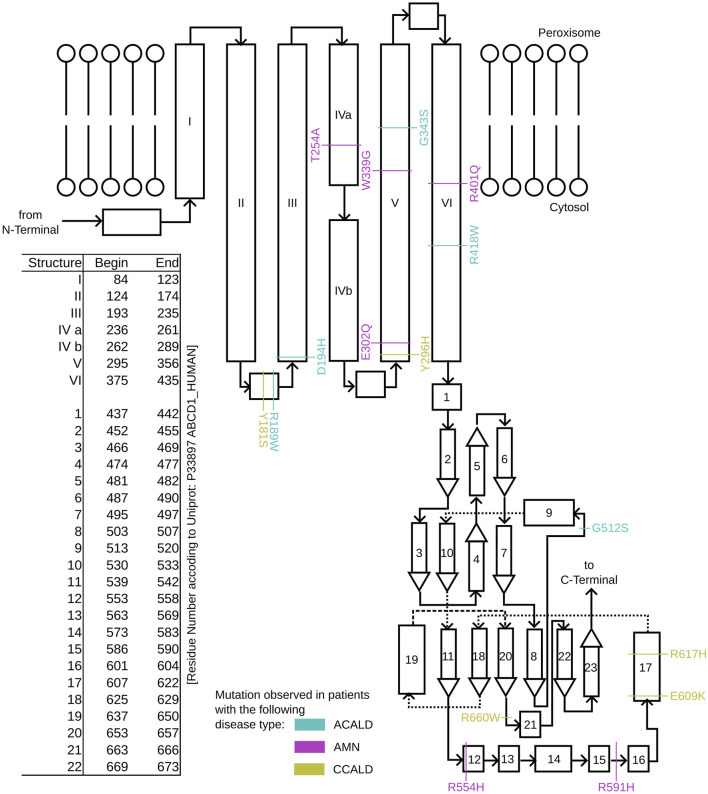
A schematic overview of the structure of single ABCD1 monomer with a list of residue positions. Positions are denoted for mutations associated with XALD disease progression in the form of ACALD (cyan), AMN (magenta), and CCALD (yellow).

**FIGURE 3:**
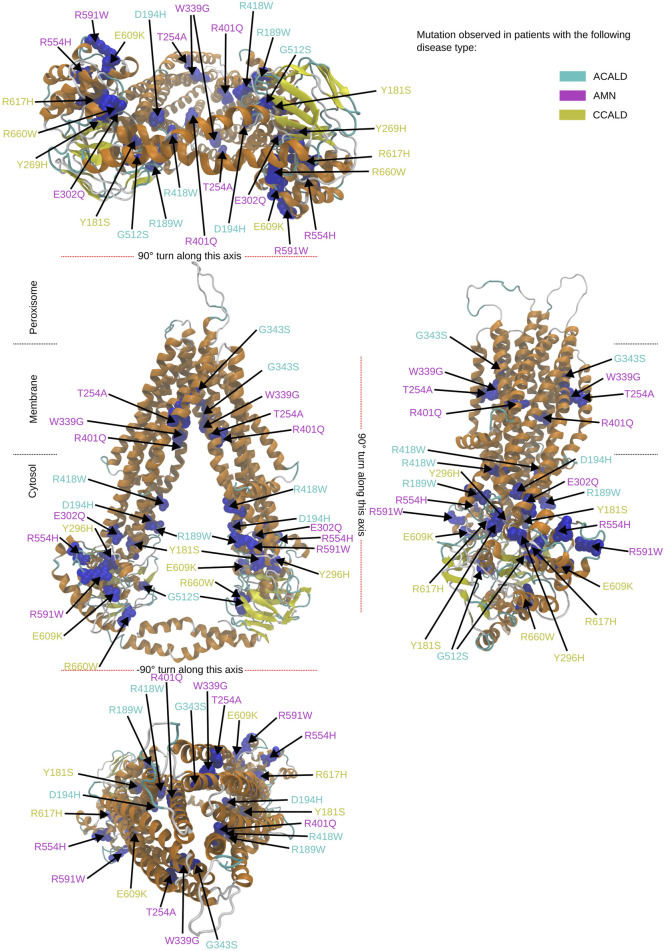
Overview of the mutated sites in our patients in the cytosol open structure. Sites to be mutated are shown as van der Waals spheres in blue. The form of disease progression is given by the corresponding color code of the residue description.

**FIGURE 4:**
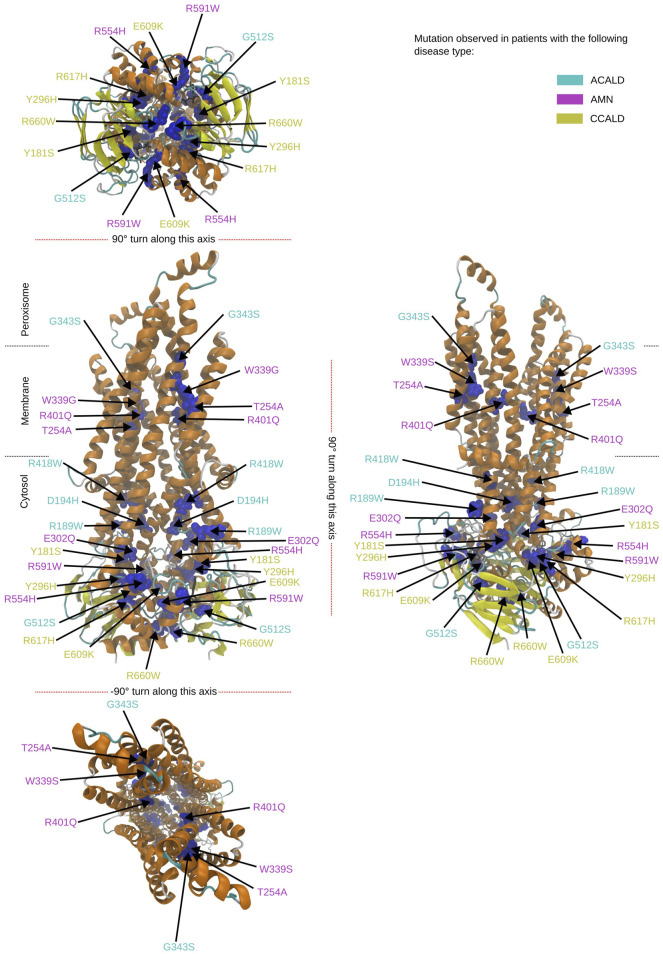
Overview of the mutated sites in our patients in the peroxisome open structure. Sites to be mutated are shown as van der Waals spheres in blue. The disease form is given by the corresponding color code of the residue description.

To study single point mutations observed in patients, a mutant was built by replacing the single amino acid for each mutation from the wild type template using MODELLER. A total of 34 structures of the ABCD1 transporter protein were generated, consisting of two structures for the wild-type (cytosol-open and peroxisome-open) and 16 derived mutations for both forms. Details about the mutations are shown in Table 1. The location of the mutations within the ABCD1 protein structure is further outlined in Figure 2 in a schematic way, while Figures 3, 4 highlight the positions of the mutations in molecular visualizations prepared using the VMD software (Humphrey et al., 1996).

**TABLE 1:** Investigated single nucleotide mutations: We outline the form of disease progression exhibited by patients when a mutation is found to affect a residue interacting with a ligand in the structure, and whether β -oxidation is affected.

Mutation	Progression type	Ligand interaction	β -Oxidation
D194H	A-CALD	VLCFA	reduced
E302Q	AMN		reduced
E609K	C-CALD	ATP	reduced
G343S	A-CALD	VLCFA	unknown
G512S	A-CALD	ATP	unknown
R189W	A-CALD		reduced
R401Q	AMN	VLCFA	reduced
R418W	A-CALD		reduced
R554H	AMN	ATP	reduced
R591W	AMN		reduced
R617H	C-CALD		reduced
R660W	C-CALD		reduced
T254A	AMN	VLCFA	reduced
W339G	AMN		unknown

### 2.2 Molecular simulation

In the previous section, 34 structures were modeled and to further analyze these structures, molecular dynamics simulations were conducted. Each simulation was set to run for 100 ns using the GROMOS force field, specifically its 56a4 parameter (Schmid et al., 2011) set, to govern the potential in the simulation. To increase computational efficiency, electrostatic interactions were evaluated using the particle mesh Ewald algorithm (PME) (Darden et al., 1993). The GROMACS software (Abraham et al., 2015) suite was utilized to prepare and execute the simulations.

Each of the 34 structures was immersed in a phosphatidylcholine (POPC) membrane with approximately 1065 POPC molecules surrounding the protein after removal of any steric clashes with the protein. For simulations using cytosol open ABCD1 transporters, a simulation box size of 20 × 19.6 × 15 ${nm}^{3}$ was prepared, while simulations starting with ABCD1 transporters in a peroxisome open conformation used a box size of 20 × 19.6 × 20 ${nm}^{3}$ . Any void areas in the simulation box were filled with water molecules, and chloride ions were added as needed to balance the positive charge of the transporter and create a neutral charged simulation box. In the peroxisome open conformations, an ATP molecule with a bound magnesium ion was included.

To prepare for the simulations, the steepest descent implementation of GROMACS was used to minimize the box configuration until the maximum force reached less than $10^{4}\frac{kJ}{mol,nm}$ . This minimized configuration was then used as the starting point for the equilibration phase. During equilibration, position restraints were enabled for the unified atoms of the modeled ABCD1 protein and, if available, for the ATP molecule. POPC and water molecules were allowed to move freely during this phase, which lasted for 30 ns to ensure proper binding of the membrane to the modeled ABCD1 protein. To increase stability and avoid oscillations, the system coupled barostat and thermostat were modeled after the Berendsen et al. (1984) type. Additionally, the Boltzmann velocity distribution was used to generate initial velocities of the simulated particles from four different seeds to the pseudo-random number generator for configurations containing the wild type of the protein, while only a single seed was used for mutated protein configurations. The barostat aimed at a reference pressure of 1 bar, while the thermostat assured a temperature of 310 ° Kelvin.

The final frames of the 30 ns equilibration trajectory were used as input for the simulation run, which utilized the more physical velocity rescaling (Bussi et al., 2007) algorithm as a thermostat, while pressure coupling was performed using the Parrinello and Rahman (1981) algorithm. The system pressure was maintained at 1 bar, with the system temperature set to 310 ° Kelvin. A 100 ns trajectory was created from each simulation for the 34 modeled ABCD1 molecules. Furthermore, six additional 100 ns simulations were conducted for the wild type configuration from differently initialized equilibrations, as outlined above. This totaled in 8 (two included in 34, plus 6) simulations, with four for the cytosol and four for the peroxisome open configuration in wild type form. Eight wild-type simulations were conducted to evaluate if simulations starting from identical conformations explore consistent conformational spaces, indicating a roughly similar exploration pattern. The study aimed to determine whether variations in conformational space exploration were independent of the initial velocities assigned to the simulation particles. Our equilibration procedure did not include a gradual relaxation of the positional restraints on protein atoms before the 100 ns production simulation. This might have led to artificial behavior of the protein in the initial stages of the production run as it adapted to its environment. Nonetheless, we maintain confidence in our simulations, which focus on mutations observed in ABCD1 patients, offering insights into the conformational dynamics of the transporter. This study serves as an important example of our tree-based conformational clustering method in action. A video of the wild type simulation is found in the Supplementary Material.

### 2.3 Basic trajectory analysis

Basic trajectory analysis was conducted on each trajectory using GROMACS tools to calculate the root mean square fluctuations (RMSFs) for the protein chains’ carbon- α atoms. This analysis provided a per-residue estimate of flexibility and allowed us to identify flexible areas of the ABCD1 transporter. To establish a baseline, the deviation of the four wild-type simulations was calculated and plotted in blue on individual graphs, while the mean of the wild-type simulations was plotted in yellow on the same graph. The RMSF of a mutated form of the protein was then plotted in red in the same figure. Regions with altered flexibility were identified by comparing the red and blue curves.

### 2.4 Protein-ATP interaction potential

During the simulations, we sampled the energy potential of the interaction between the ATP molecule and the amino acids within a 5 $\overset{{}^{\circ}}{A}$ range from the ATP molecule. This potential is the sum of long and short-range Lennard-Jones and Coulomb potentials as specified in the GROMOS force field (Schmid et al., 2011). Our focus was on understanding how the mutations in the ABCD1 protein affect the Protein-ATP interaction and whether they render the transporter non-functional.

### 2.5 Principal component analysis

The trajectory data obtained from the simulations was analyzed using principal component analysis (PCA), with the GROMACS suite of tools. Only the carbon- α atoms present in the trajectories were considered for this analysis. As we investigated only single point mutations of the protein, all carbon- α trajectories had the same number of degrees of freedom. The trajectories from the various mutations were concatenated with the data from a single simulation of the wild type, and the individual trajectory frames were sterically aligned to each other before PCA was performed. 10 eigenvalues and their corresponding principal components (eigenvectors) were retained, which spanned a 10 dimensional subspace. These 10 dimensions capture approximately 73% of the information from the carbon- α atom movements in the molecular dynamics trajectory, as indicated by the ratio of the sum of the top 10 eigenvalues to the sum of all eigenvalues, which is ≈ 0.726. It is important to note that the treebuilding algorithm, being based on DBSCAN, may not perform optimally in extremely high-dimensional spaces. Therefore, a balance needs to be struck between dimensionality reduction and maintaining meaningful clustering results. This 10 dimensional subspace was used to project the concatenated trajectory consisting of the wild type simulation and the 16 mutations. Additionally, a concatenated trajectory of only the carbon- α atoms from all the wild type simulations was created and projected into the same 10-dimensional subspace. By demultiplexing the PCA data according to mutations and disease type, individual frames could be identified and assigned to specific mutations and diseases. Apart from analyzing all carbon- α trajectories, PCA was also performed on a subset of carbon- α atoms. Residues involved in the VLFCA transport from the cytosol outward configuration were selected, and PCA was performed using the same protocol as before. Finally, residues within 5 $\overset{{}^{\circ}}{A}$ of the two ATP molecules were selected, projected, and PCA was performed in a similar manner. These non full protein PCAs did however not yield any conclusive results.

### 2.6 Tree building algorithm

To effectively distinguish between different disease types/stages, we employed an adaptive clustering approach on ensembles of conformations in the 10-dimensional subspace defined by the principal components. The underlying idea is that, during molecular dynamics simulations, prevalent conformations tend to cluster around local minima, which are mutation- and disease-specific. To correctly identify these local minima, we used the DBSCAN algorithm and scanned adaptively for clusters at different densities. The minimum density, $\rho_{\text{min}}$ , was determined by a given radius, r , and the number of conformations, n , to be searched within a hypersphere with such a radius. Ensembles featuring a connected region whose density $\rho >\rho_{\text{min}}$ were identified. By increasing $\rho_{\text{min}}$ , we were able to find more connected ensembles that intrinsically contained disconnected clusters from a previous run, allowing us to study the entire 10-dimensional PCA space and discern clustered ensembles of protein structures and local minima inside it. Parts of MNHN-Tree-Tools (Haschka et al., 2021) were used for this purpose. Figure 1 illustrates the algorithm that enabled us to efficiently find potential wells and structures associated with specific ABCD1 mutations and related X-ald disease forms observed in our patients. The algorithm has three parameters, as explained in detail in MNHN-Tree-Tools: $\epsilon_{\text{init}}$ , the initial radius; Δϵ , the radius increase in each step; and minpts , the number of samples to be found within the radius. We considered only the $L_{2}$ -Norm distance measure herein, even though MNHN-Tree-Tools provides for distance measures of all kinds. Four different trees were built corresponding to the PCA analysis above: for the peroxisomal open structure, one considering all carbon- α atoms and one considering the carbon- α atoms of the residues within 5 $\overset{{}^{\circ}}{A}$ distance from the ATP molecule; for the cytosol open structure, one considering all carbon- α atoms and one considering only the carbon- α atoms of residues that interact with VLFCA molecules. The parameters for constructing the tree were derived initially by examining the outcomes of 2D principal component analysis. This analysis provided a rough estimation of distances between points, enabling a preliminary selection of the descent radius, ϵ . Subsequently, a manual bisection process was employed on the initial ϵ parameter to determine the optimal cluster count. If the resultant trees exhibited too few branches, the Δϵ value was reduced to generate trees with greater complexity and more intricate structures. The parameters are outlined in Table 2. Once the tree was built, it was colored according to different disease progression subtypes (A-Cald, AMN, and C-Cald), and we further created a coloration discerning mutations involved with ATP binding or not. The resulting tree for simulations starting with a cytosol open conformation is outlined in Figure 9. As every node in the tree corresponds to a cluster of protein conformations visited during the simulation, we can identify conformations that are only accessible for a certain disease progession type. We can further find conformations that are typical for the wild-type protein and that might be inaccessible for a mutated protein.

**TABLE 2:** Parameters were used for adaptive clustering within a 10-dimensional subspace spanned by principal components using MNHN-Tree-Tools.

	$\epsilon_{\text{init}}$	Δϵ	minpts
peroxisome open, all C- α	0.83	0.01	4
peroxisome open, ATP pocket C- α	0.135	0.001	4
cytosol open, all C- α	3.7	0.05	4
cytosol open, VLFCA pocket C- α	0.3	0.001	4

## 3 Results

### 3.1 Results from visual inspection and mutation mapping

The structures 7vwc and 7vx8, as reported in the Protein Data Bank (PDB) (Berman et al., 2000), were visually inspected to identify the locations of mutations found in our in-house patients (Chen et al., 2022). It was observed that the majority of mutations associated with the C-CALD form were located in the ATP binding cassette domain, while the adult form AMN and ACALD associated mutations were distributed along the transporter. Visual representations of the protein structures with the identified mutations in our patients can be found in Figures 3, 4. The VMD program has been used in order to visually inspect molecular dynamics trajectories and provide 3D rendered visualizations (Humphrey et al., 1996).

### 3.2 Protein movement and stability

Simulations of cytosol open conformations revealed substantial collective movements of the proteins. A movie of a cytosol open simulation is shown in the Supplementary Material. These findings suggest that the wide-open conformation observed in cryo-electron microscopy (cryo-EM) experiments may not exist under natural conditions. Upon visual inspection of the simulation trajectories, a collapse leading to the collision of the two ATP-binding cassettes was observed. However, the transmembrane helices, particularly those embedded in the membrane, appeared to be minimally affected by this collapse. The internal spaces within the protein remained conserved, which suggests the potential for VLCFA binding.

**FIGURE 5:**
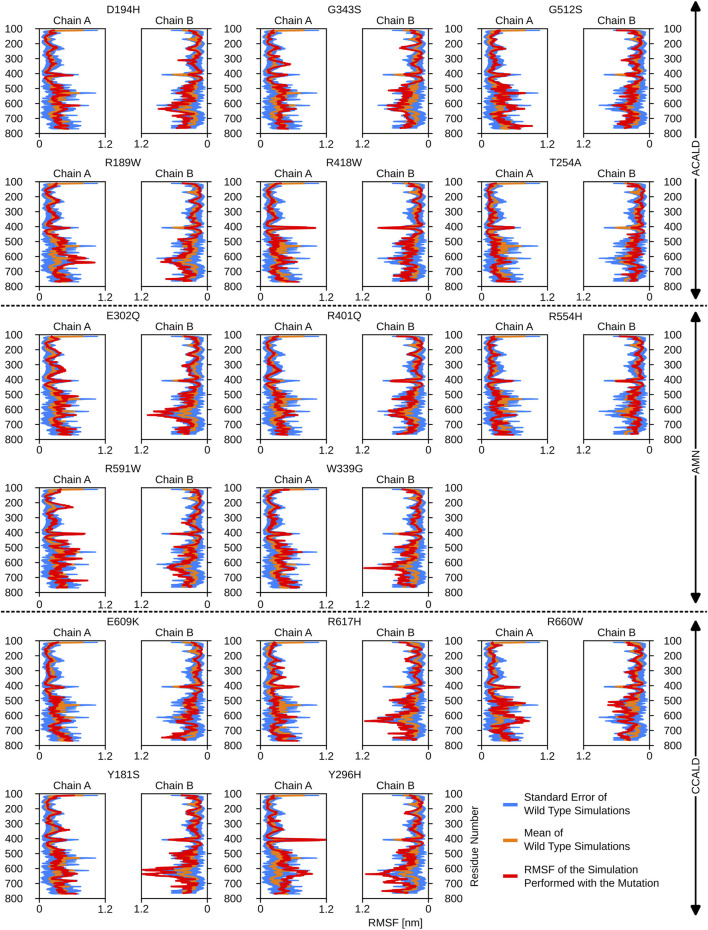
The root mean square fluctuations for the simulations performed beginning with a cytosol open structure.

Interestingly, mutations associated with the C-CALD progression form of the disease exhibited enhanced movements in the ATP-binding cassette, with the exception of E609K and R660W as shown in Figure 5. Figure 5 further reveals that there are variations in local flexibilities between the monomers of the protein. This could be attributed to the intricate allosteric mechanisms found in ABC transporters, as observed in studies by Acar et al. (2020). Notably, strong flexibilities were observed in transmembrane helix VI encompassing residues 400 to 420, which included mutated residues found in our patients. Specifically, the R418W mutation increased local flexibility within this region. Intriguingly, during the simulation of the Y296H mutated protein, this section displayed significant flexibility, underscoring the importance of the neighboring transmembrane helix V in establishing the proper interlinkage between the transmembrane helices and the ATP-binding cassette.

**FIGURE 6:**
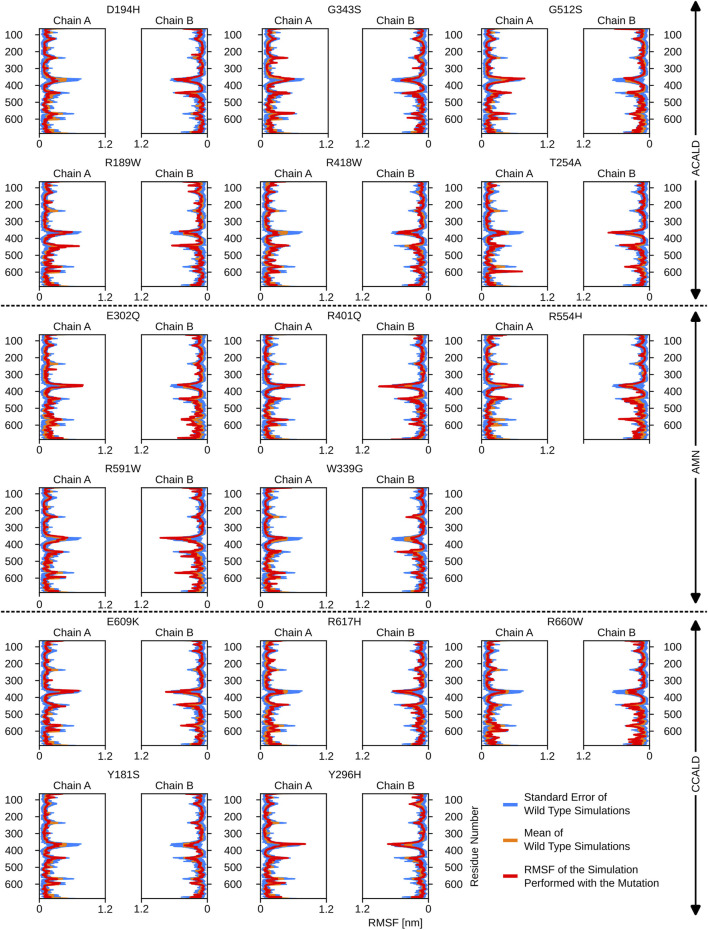
The root mean square fluctuations for the simulations performed beginning with a peroxisome open, ATP bound, structure.

Furthermore, the region between residues 600 and 700, particularly within the ATP-binding cassette, exhibited notable flexibility, especially in cases of C-CALD forms. Detailed information regarding these observations can be found in Figure 5. In this section, we emphasize residue R660 and the observed mutation R660W. As illustrated in Figure 4, R660 interacts intermolecularly, with itself. The effects of this mutation are profound across various aspects, including localized fluctuations and stability (Figures 5, 6), as well as deviations in ATP binding energy (Figure 1).

### 3.3 ATP-binding energies in peroxisome open simulations

By including ATP molecules in our molecular dynamics simulations, we were able to assess the force field energy between ATP molecules and residues within a 5 Å distance from them. The binding energy in this investigation primarily consists of the sum of the non-bonded terms of the GROMOS force field. Interestingly, mutations in the transmembrane helix appeared to affect ATP binding interactions. More strikingly the link between transmembrane helix I and II seems to play a crucial role in ATP-binding stability as we observe shifts in the ATP-binding energies in simulations of the mutated proteins D194H or Y181S.

Simulations involving mutations of residues that directly interact with the ATP molecule, such as R591W, E609K, and G512S, also exhibited significant deviations in binding energy, as expected. The mutation R660W, that can locally interact with itself as shown in the peroxisome open conformation, (c.f. Figure 4) also showed a noteworthy deviation in binding energy.

**FIGURE 7:**
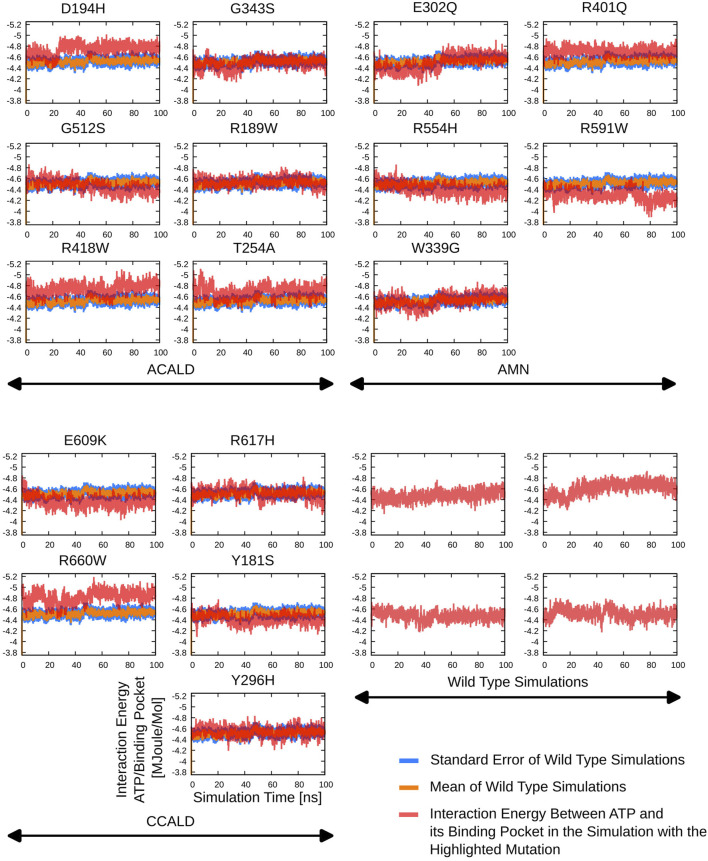
The interaction potential as evaluated during the different simulations starting from peroxisome open simulation between the ABCD1 dimer complex and two bound ATP molecules.

Detailed results of the ATP-(residues within 5 Å) interaction energies are outlined in Figure 7.

### 3.4 Results from PCA and tree-based subspace sampling

#### 3.4.1 Classical PCA result

**FIGURE 8:**
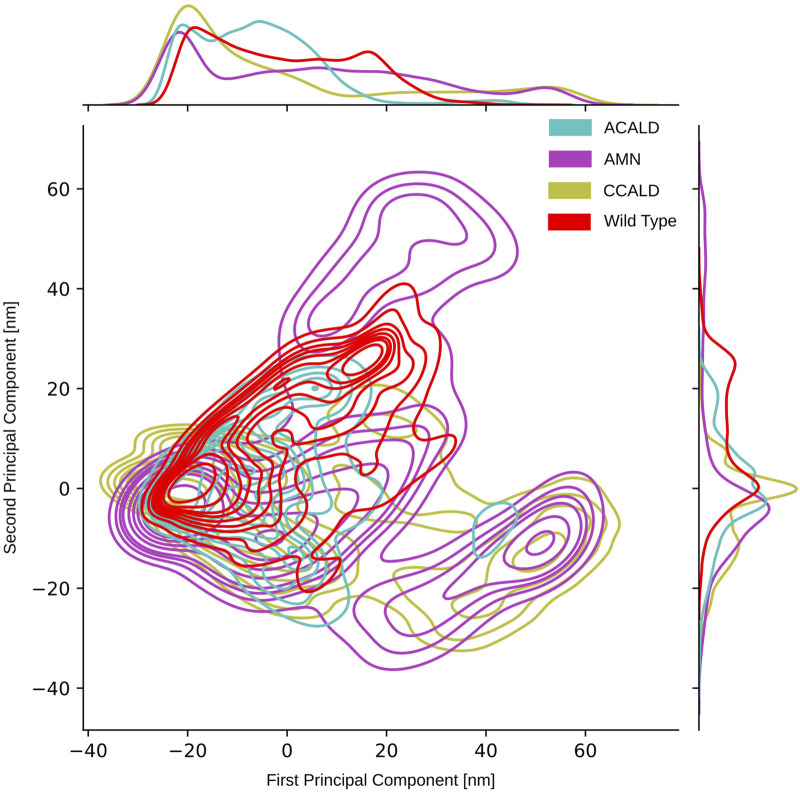
PCA performed on the carbon- α atoms of simulations that have been initialized with the cytosol open conformation.

As described in Section 2.5, we conducted principal component analysis (PCA) on the spatial coordinates of the carbon- α atoms of the simulated proteins. The PCAs performed on simulation trajectories originating from the ATP-bound peroxisome open starting point did not yield significant results due to the absence of large collective motions. Conversely, cytosol open simulations, which exhibited substantial collective movements as discussed in Section 3.2, demonstrated that the two ATP binding cassettes approached each other. Figure 8 displays a two-dimensional representation of the PCA obtained from trajectories initiated from a cytosol open starting point. As expected, this figure illustrates a confined space for the wild type simulations, while simulations with diverse mutations explore a broader conformational space.

To visually analyze the distribution of conformations generated by simulations of mutations corresponding to different disease forms, we color-coded them accordingly. In our PCA projection, we observed that mutations associated with the peripheral AMN form occupied the largest space, followed by C-CALD and A-ACALD regions. Notably, the PCA appeared to consist of two distinct densely clustered islands. The larger island on the left of the image was sampled by all forms of disease progression as well as the wild type, and different disease forms exhibited overlapping areas with varying densities on this island. Additionally, in Figure 8, we identified an island in the lower right region that comprised protein conformations exclusively accessible through mutated proteins. However, this island contained conformations from simulations of mutations found in A-CALD, AMN, and C-CALD patients. A-CALD conformations were observed in smaller quantities on this island, and the maximum concentration of A-CALD conformations appeared slightly shifted to the right, while the distributions of AMN and C-CALD overlapped around a dense central spot.

#### 3.4.2 PCA and tree-based conformational sampling

To overcome the limitations of two-dimensional PCA and further explore the conformational space, we employed a tree-based sampling approach as described in the introduction and Section 2.6. This approach enabled us to identify clusters of various densities, allowing us to uncover local minima in the energy landscape. During MD simulations, conformations close to and within these minima are frequently visited, resulting in clusters of densely packed conformations in space. The adaptive nature of our approach facilitates the merging of energy minima separated by lower energy barriers into common clusters more rapidly than those separated by larger energy barriers. Moreover, the algorithm not only provides a tree-like visualization of the sampled energy landscape but also enables exploration of separated islands in a higher-dimensional subspace of principal components that may not be visually accessible in 2 or 3 dimensions.

By applying this approach, we successfully separated the PCA island mentioned earlier, located in the lower right section of Figure 8 and described in detail previously. We were able to clearly distinguish clusters of conformations belonging to different disease forms, facilitating investigation of the distinct conformations within these clusters.

**FIGURE 9:**
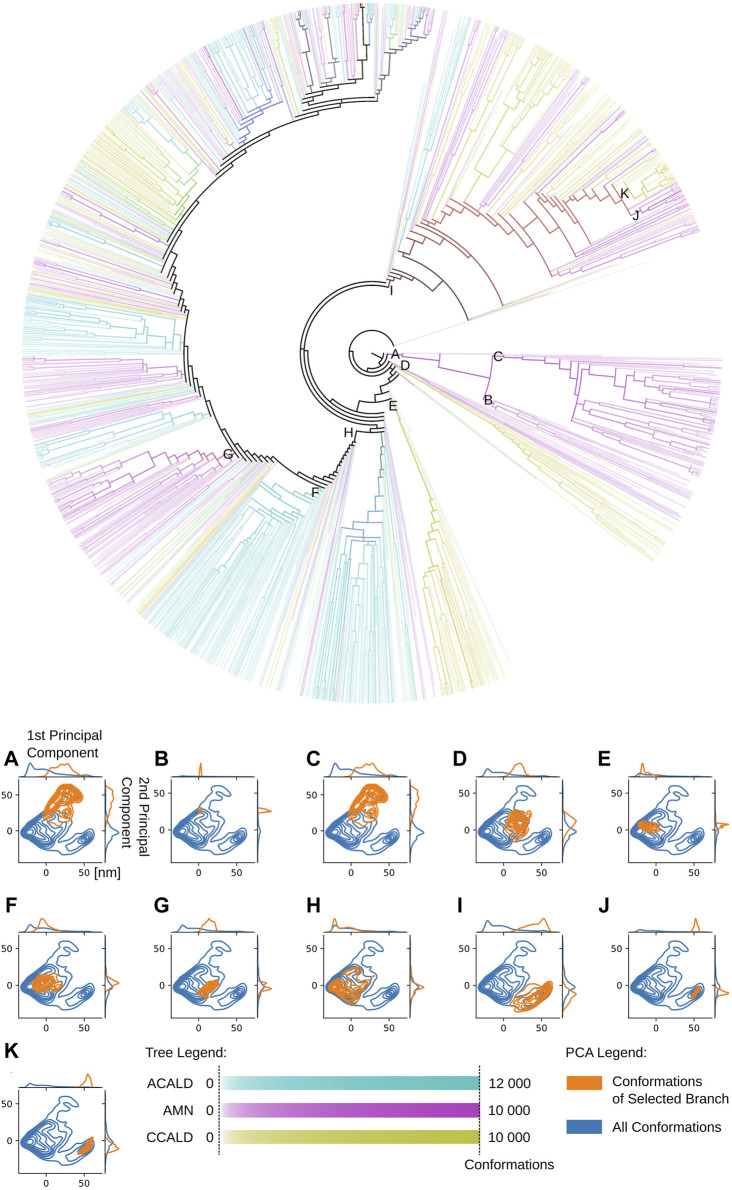
A tree view of the conformations of our simulations. Dense clusters are found on the outside of the tree. Diffuse, less dense and more connected clusters on the inside. Colors as well as overlapping colors represent the disease progression forms associated with the conformations in the cluster according to its mutation/simulation. The intensity of the color logarithmically expresses the number of conformations, from white for at least 5 conformations to full color intensity for all conformations found of the specific type. The formula used to determine the intensity of a color associated with a specific disease type is given by: $I=\frac{\log_{10}\left(conformations\;in\;a\;node\right)}{\log_{10}\left(all\;conformations\right)}$ . PCA diagrams **(A–K)** correspond to tree branches **(A–K)**.

An illustrative example is presented in Figure 9. This figure showcases a tree representation created from a 10-dimensional PCA. Different colors on the tree correspond to the three disease progression forms, C-CALD, AMN, and A-CALD. Each branch in the tree represents a cluster of a specific density, with dense clusters located on the outer portions of the tree and diffuse clusters situated toward the inner regions. Annotations A-K in Figure 9 highlight specific clusters. Additionally, the figure includes small images associated with each annotation (A-K), where the blue cloud represents the entire dataset and the orange cloud highlights the tree branch corresponding to the respective annotation in the tree.

**FIGURE 10:**
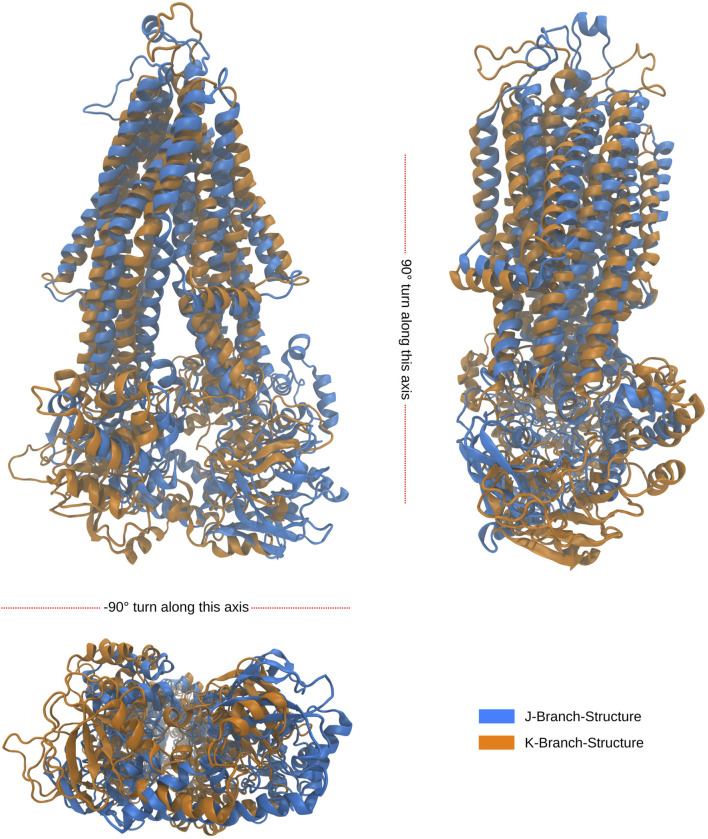
Alignment of folds of the latest, in simulation time, occurring structures from the J and K branches at outlined in Figure 9. The root mean square deviation (RMSD) between the backbone atoms of the two structures is recorded to be 1.824 nm. The J structure is representative of a cluster of 741 over 34,000 structures, while the K structure is representative of a cluster of 576 over 34,000 structures. Both clusters were found to be a density connected ensembles of structures satisfying $\rho \left(J,K\right)>\frac{480}{\pi^{5}R^{10}}$ for R=5.1nm in the 10 dimensional PCA subspace.

Notably, the tree-based representation clearly separates the two islands observed in the PCA: the larger one on the left and the smaller one in the lower right. This separation is well explained by branches H and I in the tree. Furthermore, branches J and K, which represent denser clusters and deeper local minima embedded within branch I, exhibit distinct characteristics. In contrast, the 2D PCA representation at the bottom of the figure demonstrates the difficulty of separating these two clusters using classical two-dimensional visualization methods. However, the tree representation in a higher-dimensional space demonstrates the visible separation of clusters J and K, providing evidence of the utility of our approach. Moreover, we demonstrate that branches J and K correspond to different disease progressions, with J representing structures generated during simulations of proteins harboring mutations observed in our AMN patients and K representing mutations observed in the disease progression form that already severely effects children (C-CALD). Furthermore, we illustrate that both branches exhibit distinct structures with different positioning of the ATP binding cassettes, as outlined in Figure 10. Overall, this tree-based algorithm offers a novel method to sample the conformational space generated by molecular dynamics simulations and we have effectively demonstrated its utility in this study.

#### 3.4.3 Data projection on trees and data analysis

Our approach using tree-based data sampling allows us to investigate the relationship between various conformations and different protein properties. To illustrate this capability, we project diverse disease progressions onto the tree depicted in Figure 9. This visualization helps us pinpoint distinct branches corresponding to varied protein structures, potentially influencing different forms of disease progression. Figure 9 was annotated by quantifying the number of conformations within each cluster node derived from molecular dynamics trajectories featuring mutated proteins associated with specific disease forms. The color scheme employs a logarithmic scale ranging from white to full intensity (all conformations of this disease-type regrouped in one cluster), highlighting different disease forms. Cyan, magenta, and yellow hues were selected to represent overlapping branches where conformations from multiple disease forms are found to superimpose in the same cluster.

**FIGURE 11:**
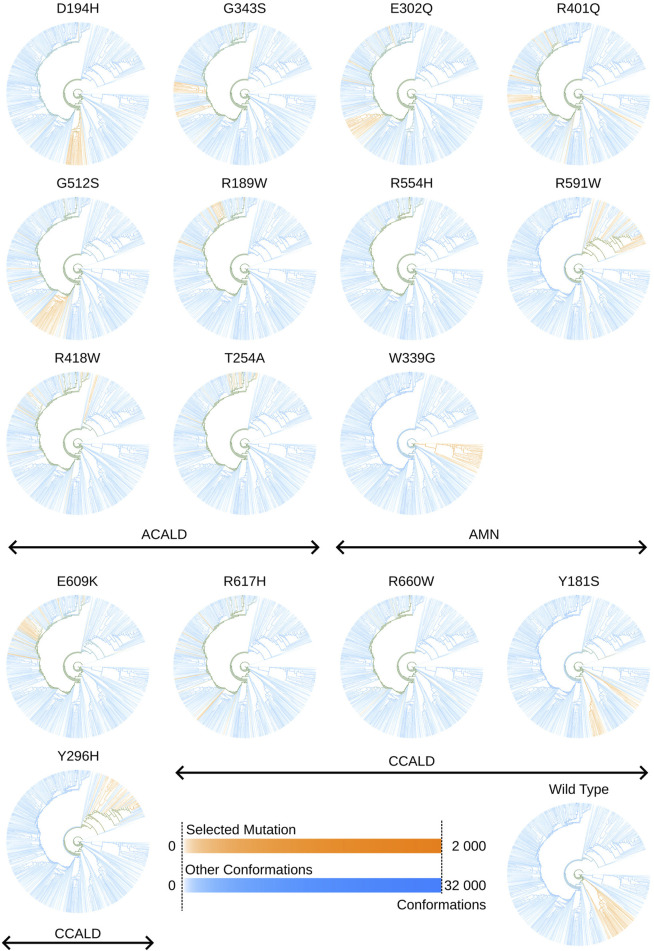
Tree views outlining in orange conformations sampled by cytosol open simulations made with a specific mutation. Colors may overlap in mixed clusters. The formula used to determine the intensity of a color associated with a specific selected mutation or other conformations is given by: $I=\frac{\log_{10}\left(conformations\;in\;a\;node\right)}{\log_{10}\left(all\;conformations\right)}$ .

We utilize this capability to explore the unique conformations generated by each simulation and mutation. Figure 11 displays trees corresponding to individual simulations, depicting clusters of conformations produced by simulations of specific mutations.

In Figure 11, two distinct colors, orange and blue, are employed. The orange color represents conformations derived from molecular dynamics simulations of the mutations under investigation, while blue denotes all other conformations within the tree. Areas where these colors overlap indicate mixed clusters. Similar to previous visualizations, a logarithmic scale ranging from white to full intensity is used for both sets of conformations: those resulting from simulations of the highlighted mutation in orange and those from all other simulations in blue.

**FIGURE 12:**
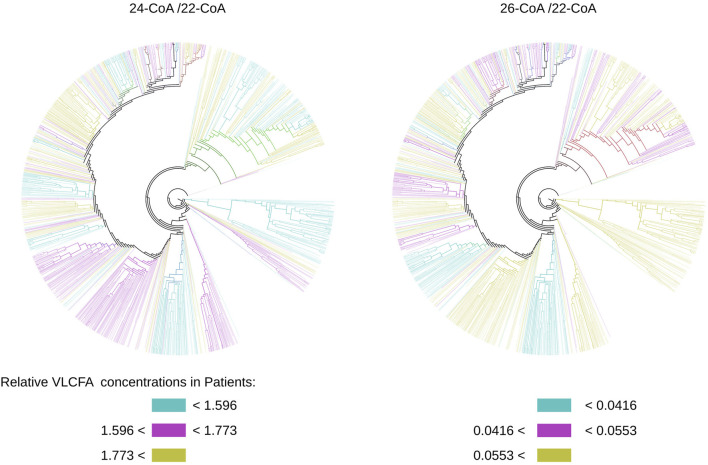
Trees built from conformation state densities for simulations starting with the cytosol-open initial condition colored by relative concentrations of VLCFA molecules observed. Colors may overlap in mixed clusters.

Additionally, this approach enables us to attribute functional properties to the protein. One crucial factor in X-ALD diagnosis is the relative concentration of very-long-chain fatty acids (VLCFAs) in the cell. In our patient data, we evaluated the concentrations of 24-C:COA and 26-C:COA relative to the concentration of 22-C:COA. By projecting these relative concentrations (24/22 and 26/22) onto our tree, we created two colored trees based on the terciles observed in our patients’ data, as depicted in Figure 12. This allows us to visually compare these trees and investigate whether the disease form C-CALD, AMN, and A-CALD (shown in Figure 9) correlate with the relative VLCFA concentrations shown in Figure 12. We observe a certain level of correlation, particularly in the case of 26/22, where A-CALD roughly corresponds to relative concentrations in the lower tercile, AMN to the middle tercile, and C-CALD to the upper tercile. Ultimately, this feature enables us to identify protein conformations that align with specific relative VLCFA concentrations.

Overall, our tree-based approach provides valuable insights into the relationship between protein conformations and various protein properties, allowing us to analyze and interpret the data in a meaningful way.

## 4 Discussion

In the pursuit of comprehending the dynamic behaviors of the ABCD1 protein and the nuanced impacts of mutations, we introduced a novel tree-based sampling method specifically tailored for molecular dynamics (MD) simulation trajectories. Traditional analyses based on static protein structures offer a snapshot view, prompting us to seek a more dynamic and inclusive approach.

Our tree-based method capitalizes on the intrinsic tendency of protein structures to aggregate in local energy minima during MD simulations, forming dense clusters of closely related conformations. This unique representation enables us to sample these clusters at various densities, constructing a hierarchical structure that reflects the energy landscape. Notably, the merging of energy minima separated by low energy barriers occurs rapidly, presenting a distinct advantage. We were further able to outline how we could separate conformation ensembles identified by the algorithm that are indistinguishable in a plain 2D PCA diagram.

To illustrate the efficacy of our tree-based method, we applied it to the study of X-linked adrenoleukodystrophy (X-ALD) and its associated mutations in the ABCD1 transporter. By incorporating this innovative approach into our analysis, we gained a dynamic perspective on how mutations influence the behavior of the ABCD1 protein. The tree-based method facilitated the correlation of functional relationships with the explored conformational space, revealing intricate insights into the structural implications of X-ALD mutations.

In conclusion, our study presents a novel tree-based sampling method that enriches our understanding of protein dynamics, outlined here in the context of disease-progression-associated mutations. The application of this method to the study of X-ALD mutations in the ABCD1 transporter exemplifies its versatility and potential in uncovering comprehensive insights into molecular dynamics. The tree-based overview of various conformations should enhance molecular docking and drug design studies by facilitating the selection of the precise molecular conformations required as input for these processes. This approach holds promise for further exploration in diverse proteins and diseases, providing a valuable tool for researchers engaged in molecular simulation studies.

## Data Availability

The datasets presented in this study can be found in online repositories. The names of the repository/repositories and accession number(s) can be found below: http://thomas.haschka.net/abcd1-simulation-data.tar.xz.

## Appendix: a practical guide to tree building using GROMACS and MNHN-Tree-Tools

Creating a tree using the method described herein does not require new code development. Instead, it leverages a combination of MNHN-Tree-Tools (Haschka et al., 2021) and the tools provided by GROMACS (Abraham et al., 2015). The process outlined below enables the construction of a tree from a molecular dynamics trajectory, highlighting the most frequently visited conformational states. In general trees are built as follows.

1. Principal Component Analysis: Initially, GROMACS tools are employed to compute principal components from a molecular trajectory. In this study, only carbon- α atoms were analyzed, although any type of trajectory could be utilized more generally. For the figures presented here, a concatenated trajectory comprising various simulated mutations was generated. Therefore, careful attention is paid to frame numbers because different frames in the trajectory can represent conformations from different mutations. Concatenation is feasible due to our focus solely on carbon- α atoms. The PCA analysis using GROMACS typically involves successive application of the *gmx covar* and *gmx anaeig* tools. Once the principal components are derived, the trajectory is projected onto a selected subset of these components. Specifically, for the trees depicted, the analysis utilized the top 10 components corresponding to the largest eigenvalues. The resulting projections are provided by GROMACS tools in the form of *xvg* files.
2. Preparing the output for MNHN-Tree-Tools: MNHN-Tree-Tools was originally designed for constructing phylogenetic trees from gene sequences, utilizing specific formats. To adapt the PCA results obtained from GROMACS for use with MNHN-Tree-Tools, we developed a small conversion script in C, which is included in the Supplementary Material of this article (*process_projections.c*). To associate each frame of a trajectory with its corresponding node in the tree, we generate a virtual FASTA file. Each sequence in this file represents a frame in the trajectory, labeled sequentially (i.e., > *frame_11950*). These labels ensure alignment with the frame numbering in the trajectory file and eliminate the need to modify MNHN-Tree-Tools’ interfaces. The sequences themselves are arbitrary (e.g., represented by the single letter ’A’), as their content does not affect subsequent calculations. Once this virtual FASTA file and the PCA data are correctly formatted, tree construction can proceed seamlessly.
3. Treebuilding with MNHN-Tree-Tools: To construct the tree, we utilized the *adaptive_clustering_PCA* tool from MNHN-Tree-Tools. This tool employs a density adaptive approach to explore conformational space (c.f. Figure 1), which is represented in higher dimensions by PCA. It utilizes the DBSCAN algorithm to identify density-connected regions, adjusting density parameters to initially locate densely packed ensembles of protein conformations, which serve as the outermost leaf nodes of the tree. As the algorithm progresses towards the root of the tree, it identifies more diffuse ensembles, into which the dense ensembles are integrated. This process effectively forms branches of the tree, merging from the leaves towards the root, where the most diffuse ensemble encompasses all sampled molecular conformations. The *adaptive_clustering_PCA* tool provides clusters at each level of this hierarchical tree structure. Due to the functionality of this tool it requires three input parameters: ϵ , the initial radius. Δϵ the increase in radius and hence, the increase in diffusion of the clusters found for subsequent stages of the tree, and finally a minimum number of points to be found within the radii at each stage.
4. Postprocessing of the Tree: The results from *adaptive_clustering_PCA* can be converted into a Newick-formatted dodendrogram using the *split_sets_to_newick* program. Various tools from MNHN-Tree-Tools can be used to color the tree, highlighting specific features as desired. To group frames for each mutation together, use the *split_set_from_annotation* tool. Maps for coloring are then generated with the *tree_map_for_splitset* set. These maps can be used in conjunction with the Newick dodendrogram as input to perform visualizations of the tree using Newick Utils (Junier and Zdobnov, 2010).

For further details of the tree building algorithm we refer the interested reader to the MNHN-Tree-Tools (Haschka et al., 2021) article, and its algorithmic supplement as well as manual. Finalized and annotated trees can be quantitatively analyzed with tools like *split_set_to_fasta* or *split_set_to_projections*, where the first one allows to identify the frame numbers (using the generated virtual FASTA file) corresponding to conformations in a tree node while the second yields the projections onto the principal components for the conformations in such a node.
